# A human-centered, participatory design approach to cancer research training

**DOI:** 10.3389/fpubh.2026.1799785

**Published:** 2026-05-13

**Authors:** Anagha Krishnan, Sydney Stern, Grace Padgett, DBora Schrett, Stephanie Zalesak-Kravec, Michele I. Vitolo, Tierra Johnson, Bret Hassel, Lena Hatchett, Laundette P. Jones

**Affiliations:** 1 NIH-Oxford-Cambridge Scholars Program, Medical Sciences Division, University of Oxford, Oxford, United Kingdom; 2 University of Maryland School of Pharmacy, Baltimore, MD, United States; 3 University of Maryland School of Medicine, Baltimore, MD, United States; 4 Principal Independent Consultant, Elite Healthcare Management Concepts, Sterling, TX, United States; 5 Marlene and Stewart Greenebaum NCI Comprehensive Cancer Center, Baltimore, MD, United States; 6 Loyola University Chicago Stritch School of Medicine Maywood, Chicago, IL, United States

**Keywords:** cancer research education, cancer survivors, community-engaged research training, human-centered design, participatory learning

## Abstract

Advancing translational cancer research increasingly requires meaningful engagement with patients, survivors, and communities; however, most research training models introduce community engagement later in training and frame it as a discrete competency rather than a formative dimension of scientific development. To explore an alternative approach, we developed a structured pilot training initiative designed to engage cancer research trainees early in their formation through sustained interaction with cancer survivors and advocates. Guided by human-centered and participatory design principles, this pilot was intentionally designed to foster early relational development between trainees and community members, cultivating solidarity, empathy, and bidirectional learning through sustained dialogue and shared inquiry. The initiative was implemented as a six-week virtual program combining didactic sessions, survivorship panels, bidirectional dialogue, and problem-based co-learning activities. Participants included biomedical trainees, scientists, cancer survivors, and advocates recruited through academic and community networks. Formative data were collected using pre- and post-session surveys and open-ended reflections to assess feasibility, engagement, and early learning signals. Across the pilot, participants reported increased comfort engaging across differences in expertise and lived experience, greater appreciation for survivor perspectives in research development, and improved ability to communicate cancer science in accessible language. Themes from the dialogue reflected a shared recognition among both trainees and survivors that bidirectional communication deepens understanding of cancer while also shaping how individuals perceive and experience their own identity. These findings suggest that intentionally sequencing cancer research training to foreground lived experience, dialogue, and co-learning may help cultivate relational capacities essential for ethical, participatory cancer research. Future work will focus on expanding and evaluating this model longitudinally and integrating formation-centered, human-centered design approaches within broader community-engaged interprofessional education efforts.

## 1 Introduction

Over the past decade, there has been growing recognition that advancing translational cancer research requires meaningful engagement with patients, survivors, and communities to ensure relevance, trust, and population-level impact (1–12). In response, cancer centers have invested in community-engaged research (CEnR) training and workforce development programs designed to equip researchers with skills to collaborate across academic and community settings (5, 10, 11). While these efforts represent important advances, much of the existing training infrastructure engages researchers after core professional identities and research orientations have taken shape. Moreover, community engagement is often introduced as a discrete competency or later-stage activity, rather than as a formative dimension of scientific training itself (2–6, 8, 10, 11). In addition, cancer survivors and advocates are frequently positioned as advisors or recipients, rather than as active contributors to the design and delivery of educational experiences that shape future researchers.

These gaps point to a broader design consideration in cancer research training: educational environments are often structured around content delivery and technical competency acquisition (13–15) with comparatively fewer opportunities to center lived experience, relational dynamics, and contextual realities that shape how research is understood, communicated, and applied. Human-centered design (HCD), which emphasizes empathy, co-creation, and iterative development grounded in stakeholder experience (16), offers a useful lens for reimagining how cancer research training can be designed to better align with human experience and community priorities. Engaging trainees earlier in their formation—when openness to experiential learning is high and professional identities are still taking shape—may offer a critical opportunity to address these gaps. At the same time, several cancer research training models have begun to incorporate survivor engagement opportunities, ranging from expanded exposure to clinical aspects of cancer to direct engagement with patients and advocates (4, 12, 13, 17–19), providing a foundation upon which further formative, community-embedded approaches can build.

Building on this foundation, we developed a structured pilot training initiative designed to engage cancer research trainees early in their training through sustained interaction with cancer survivors and advocates. This pilot centers on (1) fostering early trainee relationships with this group and; (2) cultivating solidarity and empathy; and bidirectional learning activities between trainees, survivors and advocates. This pilot represents an early, formative component of a broader initiative, “*From Cells to Communities* (C2C),” a human-centered, participatory training model that integrates biomedical science with lived experience to foster bidirectional learning between trainees and those impacted by cancer, which will be described in a future manuscript. Within this larger C2C framework, this pilot served as a practical extension of the model by creating intentional space for shared dialogue, reflection, and mutual knowledge exchange. It bridged biological mechanisms with lived experience and prepared trainees to engage thoughtfully in future community-engaged and participatory cancer research.

## 2 Pedagogical framework: bidirectional co-learning design

This pilot training initiative was intentionally designed by a diverse organizing team that included cancer faculty and professionals, biomedical research trainees, and cancer survivors. This team-based, participatory educational approach was used to operationalize bidirectional learning and co-learning throughout all stages of the pilot. In this context, bidirectional learning refers to the reciprocal exchange of biological and experiential knowledge between trainees and survivors, while co-learning describes the shared, dialogic process through which participants jointly explored cancer across scientific and lived perspectives (20).

### 2.1 Human-centered and participatory design framework

We used a human-centered (16) and participatory design (21) orientation that prioritized the experiences, needs, and perspectives of both trainees and cancer survivors throughout the development of this activity. Human-Centered Design (HCD) is a mindset and an approach, and this formative case study utilizes the HCD approach for participatory design. The HCD process involves five steps, Discovery, Define, Design, Prototype and Test, and Plan and Implement. Rather than applying a prescriptive curriculum model, the organizing team approached this pilot as an iterative educational prototype, shaped through ongoing engagement, reflection, and adaptation. Survivors and trainees were not treated as end-users of a finalized intervention, but as co-designers whose insights informed pilot goals, session structure, facilitation strategies, and learning activities. Consistent with human-centered design principles, empathy and lived experience served as foundational design inputs. Weekly planning discussions were held that provided everyone on the team an active voice from the formulation of goals to choice of topics and dissemination. These insights informed decisions related to session sequencing, facilitation norms, engagement formats, and the balance between didactic content and dialogue. To support ongoing reflection and iterative improvement, the organizing team held debriefing sessions following each session. These discussions addressed both process and logistics, including group dynamics within breakout sessions and challenges related to the online platform. The debriefs functioned as iterative design checkpoints, allowing the team to refine subsequent sessions in real time in response to participant experience.

## 3 Learning environment

The pilot activity included a series of six virtual 1.5-h sessions, each of which facilitated trainee-survivor interactions, including both lecture-based and bidirectional dialogue strategies (i.e., participants could interact via video, voice, or chat). Advertisement began 2 months before the pilot launch through virtual flyer distribution to various academic and survivor group Listservs which highlighted the purpose of the pilot, titles of topics for each week, and the scheduled meeting times. This pilot implemented a variety of virtual activities that provided opportunities for participants, including basic science trainees, patient advocates, and cancer survivors, to directly interact. The pilot had two aims: (1) to create a safe, virtual learning space for all participants to feel comfortable engaging in dialogue on the scientific and/or social dimensions of cancer, and (2) to explore the feasibility of using a virtual format to implement a variety of opportunities for engagement. Building off engagement strategies trialed by other groups, (4, 8, 22) we explored three different strategies of trainee-survivor engagement: traditional lecture format, bidirectional dialogue, and problem-based learning. Weeks 1–3, which were run in a lecture format, were conducted on WebEx and Weeks 4–6, which focused on bidirectional dialogue, were conducted on Zoom. A description of sessions and engagement style can be found in Table 1.

**Table 1:** Alignment of weekly virtual sessions with human-centered design phases.

Week	Topic	HCD	Description
Weeks 1–3 foundational knowledge + exposure	Introduction: Applying a Public Health Lens to Cancer	Discovery and define	Introduction to multi-level approaches to understanding cancer from both a basic science and a public health perspective was presented in lecture format
	Survivor Panel	Discovery and define	A panel of survivors spoke about their cancer journeys, their perspectives on cancer, and answered prepared questions as well as questions from the audience.
	Communicating with Cancer Patients/Survivors	Prototype and test	A scientist and survivor team spoke about their experiences developing a cancer community- academic partnership and shared their advice on fostering productive conversations between cancer scientists and cancer survivors
Weeks 4–6 application through bidirectional co-learning	Cancer 101 Speed Dating.	Prototype and test	~ 2:4 ratio of survivors: trainees were paired together to engage in two-minute trainee research talks followed by Q&A and feedback from survivors on their understanding.
	Trainee presentations	Prototype and test	Research trainees presented 15-min lay talks on their research, followed by the audience and the survivor judges Q & A and feedback on clarity and understandability.
	Group Problem Based Bidirectional Learning	Plan	Small groups of trainees and cancer survivors discussed their thoughts and experiences around complex and often poorly understood cancer-related topics.

HCD, Human-Centered Design; Q&A, Question and Answer.

### 3.1 Data collection and analysis

A pilot survey with multiple choice and open field responses through Research electronic data capture (RedCap) was developed for this pilot. At the beginning or end of each virtual lecture, the survey URL was included in the chat to prompt participants to complete the pre-survey and at the end of week 6, a post-survey was provided. The information collected for the pre and post surveys were the same, including weeks attended, self-identification as a “research trainee,” “career scientist,” “clinician,” “cancer patient/survivor,” “caregiver,” “advocate,” “both cancer patient/survivor and trainee”, “both trainee, cancer patient/survivor and caretaker” or “other.” Research trainees were defined as undergraduate, graduate, or postdoctoral students. Additionally, three open-ended questions were included in pre- and post-workshop surveys to elicit formative reflections from participants. The pre-survey asked, “What do you hope to learn from attending initiative?” The post-survey asked, “What did you learn from attending this initiative?” and “What do you feel could be improved upon for future initiatives?” Responses were collected voluntarily as part of pilot implementation and were used to generate formative feedback aligned with aims. Reflections were reviewed and manually grouped into two broad, illustrative categories based on participant perspective: scientific (trainees/scientists/clinicians) and experiential (survivors/patients/caregivers/advocates). This categorization was conducted by A. K. and reviewed by all members of the analysis team. Although the course spanned 6 weeks and included multiple forms of data collection across sessions, the analysis presented here focuses on the final week 6, where participants engaged most directly in collaborative, problem-based bidirectional learning. The data from earlier sessions were used to inform course delivery, but are not analyzed in this manuscript. Consistent with a human-centered, participatory design orientation, qualitative responses were not intended to represent exhaustive thematic analysis or formal qualitative evaluation. Rather, selected quotations are presented to illustrate participant experiences and early signals of alignment between pilot design and intended outcomes. This training activity was reviewed by the Institutional Review Board and received a determination of not human subjects’ research.

## 4 Results

The virtual platform provided an opportunity to extend advertising efforts beyond our geographical location. A total of 119 individuals initially registered to attend, and 75 registrants attended at least 1 out of the six sessions. Seventy-five percent of the registrants resided either in the greater Baltimore or greater Delaware-Maryland-Virginia (DMV) area, the other registrants were from other states in the US and one registrant was from India. Among the survey participants, the highest response was from those who identified as trainees (48.9%), next were scientists (19.1%), and then cancer survivors (17%). Notably, one trainee also identified as a survivor. Trainees and scientists held a wide range of research interests, including cancer biology (17.2%) and immunology (17.2%) ranking the highest. Survivors represented diverse cancer diagnoses, including breast cancer, thyroid cancer, and sarcoma.

### 4.1 Outcomes of the pilot: week 1 to week 3

Using a WebEx format, the first 3 weeks emphasized structured knowledge development and guided exposure. This reflects Discovery and Define phase of HCD (see Table 1). Week 1 introduced participants to multi-level perspectives on cancer, health disparities, and the human experience of cancer, as well as introductory skills for public communication of cancer science. The lecture encouraged biomedical trainees to broaden their focus beyond molecular pathways of tumorigenesis to consider the interacting biological, behavioral, social, and environmental factors that shape cancer risk, experience, and outcomes. Participants were also introduced to the concept of cancer health disparities, including the persistence of potentially preventable cancer deaths among populations that bear a disproportionate burden. They were also introduced to transdisciplinary research approaches that aim to reduce cancer burden and related disparities through coordinated action across multiple sectors (e.g., basic, clinical, translational, and public health research) and in partnership with communities.

In week 2, a panel of survivors of various cancer types and time since diagnosis, were invited to share their experiences and perspectives, including perspectives on treatment, survivorship, and cancer research. During the survivorship panel, survivors described the multifaceted challenges of cancer treatment and survivorship, emphasizing the physical, cognitive, and emotional adjustments required over time. Participants highlighted the difficulty of navigating treatment-related uncertainty and loss of control, noting that while the physical effects of surgery and chemotherapy were significant, the cognitive and emotional impacts were often more challenging (e.g., “what happened inside my head was even harder”). Survivors also described how treatment goals and definitions of success evolved across the cancer trajectory, with outcomes shifting from cure or remission to functional goals such as maintaining mobility or achieving disease stability—endpoints that are not easily captured by traditional clinical metrics (“my goal shifted from being able to run again to just being able to walk”).

Survivorship experiences were further characterized by ongoing uncertainty, fear of recurrence, and the emotional labor of living with cancer as a chronic or recurring condition. Survivors emphasized the importance of sustaining hope throughout this process, even when disease progression occurs, reframing hope as the ability to manage symptoms, maintain quality of life, or simply experience a “good day.” In discussing cancer research, survivors expressed a desire for stronger connections between researchers, clinicians, and patients, noting that patient perspectives can provide critical insight into feasibility, relevance, and priority-setting in research (“what they’re pursuing… needs to be feasible in the real world”). Survivors underscored the value of incorporating lived experience into research conversations, suggesting that such engagement could meaningfully inform research direction and ultimately improve patient-centered outcomes.

Presenters from Week 3 were representatives from the Cornell Community Cancer Partnership who shared the history of how a partnership between an academic institution and a non-profit cancer survivor organization developed a formal curriculum to expose cancer research trainees to broader social aspects of cancer and provide opportunities to experience the human side of cancer through interactions with patients and survivors (5). The lecture offered guidance on approaching conversations about cancer with patients and survivors, communicating cancer science in public settings, and fostering safe, inclusive environments for dialogue, which collectively supported trainees’ readiness for reciprocal, survivor-centered engagement in later bidirectional co-learning sessions.

### 4.2 Week 4 to week 6

Building on this foundation, Weeks 4–6 were intentionally designed to shift toward bidirectional, problem-based co-learning dialogues, in which trainees and cancer survivors engaged as equal contributors to shared inquiry and meaning-making. This reflects prototype and test phase of HCD (see Table 1). Weeks 4–6 were held using a Zoom virtual format which allowed for smaller group interactions and deeper engagement. In Week 4, trainees and survivors were paired in groups of five (3 trainees, 2 survivors) in Zoom break out rooms where trainees had the opportunity to practice presenting a three-minute summary of their research to survivors. During Week 5, selected trainee volunteers presented informational/research presentations on a variety of fields of cancer, including drug design, genetics, and immunology to a panel of judges made up of cancer survivors as well as an audience of their peers and the public. The audience and the judges had the opportunity to ask questions and advise the trainees on how to be clear and concise in their presentations in a real-time manner. Overall, participants responded favorably to both the bi-directional engagement strategies.

The most engaging and interactive session was the Problem-Based Bidirectional Co-Learning Dialogues held in week 6. This reflects the HCD Plan and Implement phase (see Table 1). These small-group sessions brought together biomedical trainees and cancer survivors to engage in shared inquiry around complex cancer-related experiences (e.g., chemotherapy-related cognitive changes), allowing biological explanations and lived experience to inform one another through facilitated discussion and reflection. This session hosted small Zoom break-out room groups to share their thoughts and experiences around several long-standing complex and often poorly understood cancer-related topics. These topics included: “All cancers are the same.”, “Cancer is caused by mutations.”, and “What is Chemo fog, a symptom experienced by cancer patients.” Within each group, trainees and scientists shared the scientific information they knew about the topic and survivors related the topic to their personal experiences with cancer. Following the small group discussions, participants shared key insights in a larger group setting. Table 2 provides an illustrative example of how one topic was “unpacked” through dialogue, integrating biomedical perspectives and lived experience. These summaries are based on facilitator notes and group-level observations and are presented as descriptive examples of the co-learning process, rather than formally defined or measured outcomes. As such, they are not derived from a formal qualitative analytic approach, but are included to demonstrate how knowledge was exchanged and meaning was co-constructed within this pilot. This unique problem solving-based engagement strategy held in week 6 was particularly well-received by both groups, with some feeling it was “a start in a place where…everyone has a common understanding of cancer.”

**Table 2:** Example of a problem-based bidirectional co-learning activity: plan and implement phase.

Activity component	Biomedical trainee perspective (biological knowledge)	Cancer survivor perspective (lived experience)	Co-learning and formative outcomes
Problem framing *“What is Chemo brain?”; Chemotherapy-related cognitive changes”*	Describes proposed biological mechanisms, including neuroinflammation, oxidative stress, altered neurotransmitter signaling, and emerging evidence related to blood–brain barrier disruption and cellular stress responses	Describes lived experiences of memory lapses, difficulty concentrating, and feeling cognitively “not like myself” during and after treatment	Reframes the topic as both a biological phenomenon and a lived experience, expanding how impact is understood
Dialogue and reflection	Reflects on how survivor experiences might inform future research questions and communication with non-scientific audiences	Reflects on how biological explanations provide context and validation for lived symptoms	Co-learning emerges through dialogue rather than parallel presentations
Formative learning outcome	Increased comfort translating complex biology for lay audiences; deeper appreciation of patient-centered perspectives	Increased understanding of biological processes underlying symptoms; affirmation of lived experience as meaningful knowledge	Cultivates relational readiness through reciprocity, openness, and shared meaning-making

As part of week 6, we collected formative feedback to better understand participants’ experiences with the pilot. Among trainees who attended and voluntarily shared feedback, reflections suggested increased attention to survivor perspectives in research development, greater awareness of the multifaceted challenges faced by cancer survivors, and increased comfort communicating cancer research in non-technical language (Table 3). Qualitative reflections further highlighted the challenges and growth associated with translating scientific concepts for non-specialist audiences (Table 3). Both trainees and survivors also described increased comfort engaging in informal conversations about cancer research following participation in the sessions. Survivors’ reflections, in particular, suggested that participation supported a sense of agency and meaning, including greater comfort sharing their cancer experiences and recognition of the value their stories bring to the scientific community (Table 3).

**Table 3:** Illustrative qualitative themes and exemplary participant reflections.

Themes	Exemplary quotes	
	Trainees/scientists/clinicians	Survivors/patients/caregivers/advocates
Trainee/survivor communication enhances our understanding of cancer	• “I gained a stronger understanding of what it really means to communicate with patients and make sure they are considered in research.” • “Having people from different backgrounds really gives a more complete perspective of answering all these different aspects of cancer.”	• “What we learn best is when we learn from each other.” • “We can accomplish more in partnership.” • “Keep the conversation going!” • “I was very encouraged by the young researchers in our group and their questions.” • “It was a learning experience for me and an encouraging one.”
Trainee/survivor communication affects individual’s feelings about their own identity	• “Remembering the patient helps reinvigorate my passion.” • It is humbling at the end of the day, [to] just be like, I do not know.”	• “I grew more comfortable talking about my own cancer diagnosis.” • “[I learned] the positive impact my story holds for the scientific community.”

## 5 Discussion

This pilot demonstrates the value of intentionally sequencing cancer research training in alignment with HCD, moving from Discovery and Define toward application through Prototype, Test, and Plan. During Weeks 1–3, trainees were introduced to multi-level frameworks for understanding cancer, survivorship narratives, and strategies for communicating cancer science in community-facing settings. Consistent with the Discovery and Define phases of HCD, these early sessions emphasized exposure to the social dimensions of cancer and the development of foundational communication skills, creating a shared language and preparing trainees to engage respectfully with lived experience. Prior work in health professions and translational research education has highlighted the importance of early exposure to patient perspectives and multi-level determinants of health (23, 24), yet such experiences remain limited in early-stage biomedical training.

Building on this foundation, Weeks 4–6 shifted toward application through structured, problem-based bidirectional co-learning dialogues. Through activities such as survivor–trainee research exchanges, lay research presentations, and small-group discussions centered on complex cancer-related experiences, trainees and survivors engaged as reciprocal contributors to shared inquiry. These activities reflect human-centered design principles by foregrounding relational interaction, contextual meaning-making, and iterative learning over unidirectional knowledge transfer (16).

Across the six-week pilot, the concept of *relational readiness*—the capacity to engage others with openness, humility, and respect across differences in expertise and lived experience (25)—began to surface in the qualitative feedback. While not formally measured in this study, our findings suggest its importance as a potential outcome of the bidirectional learning environments. Rather than treating community engagement as a discrete competency to be acquired later in training, our pilot positioned engagement as an orientation shaped through sustained exposure, reflective dialogue, and co-learning. The survivorship panel played a particularly important formative role by offering trainees rare insight into the emotional, cognitive, and social realities of cancer—dimensions that are often underrepresented in traditional biomedical curricula. This exposure supported empathy, openness to multiple forms of knowledge, and a more holistic understanding of cancer impact, preparing trainees to participate meaningfully in subsequent bidirectional learning activities. From a design perspective, these findings suggest that relational readiness may emerge from educational environments intentionally structured to support empathy, mutual respect, and shared authority (25, 26). As a next step, future work will aim to more explicitly define and assess the concept of relational readiness using established measures to better understand its role in shaping meaningful community-engaged practice.

This study has several limitations that should be considered. Because this formative pilot focused on cancer survivors and advocates, the findings may not extend to broader community populations, particularly those without prior exposure or lived experience, highlighting an important area for future research. An additional limitation of this study is that participant demographic data was not collected. Given that cancer health disparities was introduced as a foundational concept in the initial session, understanding participant characteristics would have provided important context for interpreting engagement and perspectives. Future work can incorporate these measures to better contextualize findings, particularly in relation to cancer health disparities.

This pilot suggests that early, community-embedded educational experiences, designed through human-centered and participatory approaches, may serve as foundational infrastructure for future community-engaged and participatory cancer research. By cultivating the capacities needed for effective engagement upstream, institutions may be better positioned to support ethical, relevant, and sustainable engagement across the cancer control continuum. Future work will focus on expanding this model, evaluating longer-term impacts on trainee development and community engagement, and integrating human-centered design principles within broader community-engaged interprofessional education (CE-IPE) (22, 27, 28, 29) initiatives aimed at strengthening the foundations of equitable and participatory cancer research. Taken together, this work points toward a formation-centered approach to research training that prepares future investigators not only with technical expertise, but with the interpersonal and engagement capacities for sustained, ethical community partnership.

## Data Availability

The raw data supporting the conclusions of this article will be made available by the authors, without undue reservation.
